# LATCH score and 6-month exclusive breastfeeding in a Baby-Friendly Hospital

**DOI:** 10.1590/1806-9282.20251466

**Published:** 2026-07-10

**Authors:** Hatice Kübra Dursun, Övgü Büke, Demet Deniz Bilgin, Mine Basibüyük, Nalan Karabayır

**Affiliations:** 1 Istanbul Medipol University, Faculty of Medicine, Department of Pediatrics – Istanbul, Türkiye.; 2 Istanbul University-Cerrahpaşa, Cerrahpaşa Medical Faculty, Department of Pediatrics – İstanbul, Türkiye.; 3 Istanbul Medipol University, Health Science Institute, Department of Social Pediatrics – Istanbul, Türkiye.; 4 University of Health Sciences, Istanbul Haseki Training and Research Hospital, Department of Pediatrics – Istanbul, Türkiye.; 5 Istanbul Medipol University, International School of Medicine, Department of Social Pediatrics – Istanbul, Türkiye.

**Keywords:** Breast feeding, Bottle feeding, Pacifiers

## Abstract

**OBJECTIVE:**

Exclusive breastfeeding for 6 months is essential for infant health. Various factors, as well as early breastfeeding performance assessed by the LATCH score, may influence exclusive breastfeeding duration, but their predictive value remains unclear. We aimed to evaluate whether early postpartum LATCH scores and other clinical or sociodemographic factors predict exclusive breastfeeding at 6 months in a Baby-Friendly Hospital.

**METHODS:**

The single-center prospective observational study was conducted at a Baby-Friendly Hospital in Istanbul, Türkiye. LATCH scores were assessed within the first 48 h postpartum by the same lactation consultant. At 6 months, follow-up interviews were conducted via phone to determine the infant's feeding status, bottle and pacifier use, and return to work.

**RESULTS:**

Of the 301 mother-infant pairs included, 54.8% maintained exclusive breastfeeding at 6 months. Higher early LATCH scores were significantly associated with vaginal delivery, previous breastfeeding experience, and exclusive breastfeeding during the first 48 h. However, LATCH scores were not significantly linked to exclusive breastfeeding at 6 months. In multivariate analysis, bottle use was strongly associated with reduced likelihood of exclusive breastfeeding at 6 months (OR 16.672, 95%CI 8.875–31.318, p<0.001).

**CONCLUSION:**

Since bottle use is the most important factor affecting exclusive breastfeeding at 6 months, preventing bottle use is a critical intervention in maintaining breastfeeding. Furthermore, the LATCH score is insufficient in predicting exclusive breastfeeding in the long term.

## INTRODUCTION

Breastfeeding is universally recognized as a cornerstone of infant nutrition, growth, and immune protection during the first 6 months of life. The World Health Organization (WHO) and the American Academy of Pediatrics (AAP) recommend exclusive breastfeeding (EBF) for the first 6 months, with continued breastfeeding up to 2 years or beyond alongside appropriate complementary foods^1,2^.

Despite these recommendations, adherence remains suboptimal. Only 41% of infants worldwide are exclusively breastfed for 6 months, well below the WHO's 2025 target of at least 50%^3^. Promoting breastfeeding could prevent over 820,000 child deaths and 20,000 maternal deaths annually, lower the risk of obesity and type 2 diabetes, and save over US$300 billion in healthcare costs and productivity losses. Breastfeeding also contributes significantly to several United Nations Sustainable Development Goals (SDGs), particularly those related to health, nutrition, and equity^4^.

In Türkiye, the 2018 Demographic and Health Survey reported that 71.3% of infants were breastfed within the first hour of life, yet the EBF rate at 6 months was only 40.7%, closely mirroring global trends^5^. Common reasons for early weaning include maternal perception of insufficient milk supply, breastfeeding difficulties, infant refusal, nipple pain, mastitis, poor infant weight gain, and maternal return to work^6–9^. Many of these challenges stem from ineffective breastfeeding techniques, highlighting the need for standardized assessment tools.

The LATCH scoring system, developed by Jensen et al., evaluates five core components—Latch, Audible swallowing, Type of nipple, Comfort, and Hold—each scored from 0 to 2 for a total score up to 10. Higher scores indicate more effective breastfeeding, but the optimal cut-off for predicting long-term success remains debated^10,11^. Several studies have found that higher LATCH scores are linked to better breastfeeding initiation and duration, especially within the first 6 weeks postpartum^12–14^. However, evidence on whether LATCH scores predict EBF at 6 months is inconsistent. Most prior studies were conducted in settings lacking standardized lactation support, and few have explored this relationship in Baby-Friendly Hospital (BFH) environments where structured counseling is routinely provided. This is one of the few studies evaluating the predictive value of LATCH scores in a BFH setting. Given these gaps, this study explores whether early postpartum LATCH scores can reliably predict EBF at 6 months, strengthening evidence for its use in BFH.

## METHODS

### Research design

The prospective observational study was conducted between March 2021 and November 2021 in Istanbul. The study was conducted at a BFH, where mother–infant dyads are routinely visited by certified breastfeeding consultant nurses at least once daily during the postpartum stay. Standard BFH practices, including immediate skin-to-skin contact, rooming-in, and avoidance of non-medically indicated bottles or pacifiers, are implemented.

The study population comprised mothers aged over 18 years who were literate, fluent in Turkish, volunteered to participate, and had no chronic illnesses. Eligible infants were full-term, had an APGAR score of ≥7 at 5 min, and had no congenital anomalies or genetic disorders. Data were collected using three instruments: (1) a Descriptive Characteristics Form, (2) the LATCH Breastfeeding Assessment Tool, and (3) a Structured Follow-Up Questionnaire administered at 6 months postpartum. A total of 330 mother–infant dyads were enrolled; after accounting for loss to follow-up, the study was completed with 301 participants. LATCH scores were assessed within the first 48 h postpartum by the same certified lactation consultant to ensure consistency.

The LATCH score, developed by Jensen et al. and validated in Turkish by Altuntas et al., was used as the standardized breastfeeding assessment tool in this study^10,15^. The tool provides a systematic method to evaluate five key components of breastfeeding technique. Each letter in the acronym represents a different domain: L (Latch)—the infant's ability to grasp the breast, A (Audible swallowing)—the number of swallowing sounds heard while the baby feeds, T (Type of nipple)—nipple type, C (Comfort)—the mother's level of breast comfort, and H (Hold)—the help the mother needs to position the baby. Each component is scored 0, 1, or 2, with a total possible score of 10. The LATCH score (0–10) was analyzed as a continuous variable in all statistical procedures. At 6 months postpartum, participants were contacted via phone and administered a structured questionnaire developed and piloted by the research team.

In the descriptive statistics of the data, mean, standard deviation, median, minimum, maximum, frequency, and ratio values were used. The distribution of variables was assessed using the Kolmogorov-Smirnov test. The Mann-Whitney U test was used in the analysis of quantitative independent data. The Mann-Whitney U test and Kruskal-Wallis test were used to compare continuous LATCH scores across clinical and demographic characteristics. The chi-square test was used in the analysis of qualitative independent data, and Fisher's exact test was used when chi-square assumptions were not met. Logistic regression was performed to determine the predictors of EBF at 6 months. Statistical Package for the Social Sciences 28.0 software was used for all analyses.

## RESULTS

### Demographics and breastfeeding practices

Of the 301 mothers included in the study, 76.1% were aged between 26 and 35 years, 62.8% held a university degree, and 54.8% (n=165) maintained EBF at 6 months. Male infants accounted for 55.5% of the sample, and 63.8% were delivered via cesarean section. Immediate skin-to-skin contact was provided in 83.1% of cases, and 74.4% of infants initiated breastfeeding within the first 30 min. The rate of EBF within the first 48 h postpartum was 88.7%. Approximately 50% of the mothers had previous breastfeeding experience (Table 1).

**Table 1:** Demographic and clinical characteristics of the participants.

		n	%
Maternal age			
	18–25 years	43	14.3
	26–35 years	229	76.1
	≥36 years	29	9.6
Maternal education			
	Primary school	35	11.6
	High school	77	25.6
	University	189	62.8
Mode of delivery			
	Vaginally (NSVD)	109	36.2
	Cesarean section (C/S)	192	63.8
First skin contact			
	Immediately after birth	250	83.1
	Within the first half hour	30	10.0
	>30 min	21	7.0
Initiation of breastfeeding			
	In the first half hour	224	74.4
	Half hour–1 h	60	19.9
	1–24 h	16	5.3
	Couldn't breastfeed	1	0.3
Nutrition in the first 48 h			
	Exclusive breastfed	267	88.7
	Other (mix feeding or only formula)	34	11.3
Antenatal education			
	Yes	51	16.9
	No	250	83.1
Previous breastfeeding experience			
	Yes	147	48.8
	No	154	51.2
Longest breastfeeding duration (month)			
	0–6	23	7.6
	7–12	19	6.3
	13–18	18	6.0
	19–24	41	13.6
	≥24	42	14.0
	Did not breastfeed	158	52.5
Starting work before 6 months after birth			
	Yes	28	9.3
	No	273	90.7
Pacifier use			
	Yes	155	51.5
	No	146	48.5
Bottle use			
	Yes	137	45.5
	No	164	54.5

NVD: normal spontaneous vaginal delivery.

The mean LATCH score was 8.5±1.2. In bivariate analyses, LATCH scores were significantly higher among mothers with vaginal delivery, previous breastfeeding experience, and EBF in the first 48 h (Table 2). No significant differences were found across maternal age, maternal education, antenatal education, starting work before 6 months after birth, the longest breastfeeding duration, first skin contact, the initiation of breastfeeding, and overall pacifier and bottle use.

**Table 2:** Relationship between characteristics and LATCH score.

		N	Mean LATCH±SD	p
Exclusive breastfeeding at 6 months	<6 months	136	8.44±1.38	0.754
	6 months	165	8.56±1.10	
Maternal age (year)	18–25	43	8.42±1.14	0.464
	26–35	229	8.50±1.25	
	≥36	29	8.69±1.28	
Maternal education	Primary school	35	8.74±1.07	0.411
	High school	77	8.58±1.21	
	University	189	8.43±1.28	
Mode of delivery	Vaginally (NSVD)	109	8.77±1.19	**0.004** a
	Cesarean section (C/S)	192	8.35±1.24	
Antenatal education	Yes	51	8.55±1.22	0.723
	No	250	8.50±1.24	
Starting work before 6 months after birth	Yes	28	8.14±1.07	0.074
	No	273	8.54±1.24	
Previous breastfeeding experience	Yes	147	8.83±1.09	**<0.001** a
	No	154	8.19±1.28	
The longest breastfeeding duration^b^ (month)	0–6	23	8.26±1.25	0.203
	7–12	19	9.00±1.10	
	13–18	18	9.00±0.97	
	19–24	41	8.88±1.10	
	≥24	42	8.88±1.04	
First skin contact	Immediately after birth	250	8.50±1.22	0.900
	Within the first half hour	30	8.43±1.40	
	>30 min	21	8.62±1.16	
Initiation of breastfeeding^c^	In the first half hour	224	8.52±1.25	0.205
	Half hour–1 h	60	8.62±1.13	
	1–24 h	16	8.00±1.15	
Nutrition in the first 48 h	Exclusive breastfeeding	267	8.57±1.21	**0.010** a
	Other (mixed feeding or only formula)	34	7.97±1.29	
Pacifier use	Yes	155	8.49±1.32	0.981
	No	146	8.52±1.15	
Bottle use	Yes	137	8.34±1.33	0.062
	No	164	8.65±1.13	

a The chi-square statistic is significant at the 0.05 level.

b This variable reflects previous breastfeeding duration with prior children, not the index infant.

c The category ">24 h/Did not breastfeed" (n=1) was excluded from the "Initiation of breastfeeding" analysis only. SD: standard deviation; NSVD: normal spontaneous vaginal delivery. Bold values indicate statistically significant results.

EBF at 6 months was significantly associated with EBF in the first 48 h (p=0.002), absence of pacifier use (p<0.001), and absence of bottle use (p<0.001). Early initiation of breastfeeding (p=0.069) trended toward positive effects but was not statistically significant (Table 3). Continuous LATCH scores were not independently associated with 6-month EBF (p=0.754).

**Table 3:** Factors affecting exclusive breastfeeding at the 6ix months.

		Exclusive breastfeeding at 6 months				
		Yes (n:165, 54.8%)		No (n:136, 45.2%)		p
		n	%	n	%	
Maternal age (year)	18–25	23	53.5	20	46.5	0.476
	26–35	123	53.7	106	46.3	
	≥36	19	65.5	10	34.5	
Maternal education	Primary school	18	51.4	17	48.6	0.731
	High school	45	58.4	32	41.6	
	University	102	54.0	87	46.0	
Antenatal education	Yes	31	60.8%	20	39.2	0.347
	No	134	53.6	116	46.4	
Previous breastfeeding experience	Yes	87	59.2	60	40.8	0.137
	No	78	50.6	76	49.4	
Mode of delivery	Vaginally (NSVD)	64	58.7	45	41.3	0.306
	Cesarean section (C/S)	101	52.6	91	47.4	
First skin contact	Immediately after birth	139	55.6	111	44.4	0.634
	Within the first half hour	14	46.7	16	53.3	
	>30 min	12	57.1	9	42.9	
Initiation of breastfeeding^b^	In the first half hour	122	54.5	102	45.5	0.069
	Half hour–1 h	38	63.3	22	36.7	
	1–24 h	5	29.4	11	70.6	
Nutrition in the first 48 h	Exclusive breastfeeding	155	58.1	112	41.9	**0.002** a
	Other (mixed feeding or only formula)	10	29.4	24	70.6	
Pacifier use	Yes	69	44.5	86	55.5	**<0.001** a
	No	96	65.8	50	34.2	
Bottle use	Yes	30	21.9	107	78.1	**<0.001** a
	No	135	82.3	29	17.7	
Starting work before 6 months after birth	Yes	14	50.0	14	50.0	0.591
	No	151	55.3	122	44.7	

a The chi-square statistic is significant at the 0.05 level.

b The category ">24 h/Did not breastfeed" (n=1) was excluded from the "Initiation of breastfeeding" analysis only. NSVD: normal spontaneous vaginal delivery. Bold values indicate statistically significant results.

A multivariable logistic regression model was performed to identify independent predictors of 6-month EBF. The model demonstrated good fit, with Model χ^2^(8)=123.936 (p<0.001), Nagelkerke R^2^=0.451, overall classification accuracy of 80.4%, and Hosmer-Lemeshow p=0.941. Mothers who used a bottle during the postpartum period showed 16.6 times higher odds of not exclusively breastfeeding at 6 months (odds ratio=16.67; 95%CI 8.87–31.31; p<0.001). Neither pacifier use (p=0.808) nor early feeding exclusively with human milk in the first 48 h (p=0.201) remained significant. Maternal age, maternal education, and previous breastfeeding duration were also not independently associated with 6-month EBF (all p>0.05) Supplementary Table 1.

## DISCUSSION

This prospective observational study investigated factors associated with EBF at 6 months and explored whether early postpartum LATCH scores could predict sustained breastfeeding. While most previous research has focused on short-term outcomes, such as EBF at 6 weeks, this study offers novel insight into the longer-term implications of early breastfeeding performance as measured by the LATCH tool.

Our findings showed that higher LATCH scores were significantly associated with previous breastfeeding experience, vaginal delivery, and EBF within the first 48 h, consistent with prior research emphasizing the importance of maternal experience and early breastfeeding practices for successful lactation^16,17^. In contrast, prenatal breastfeeding education was not linked to LATCH scores. Earlier studies have reported lower LATCH scores among mothers lacking prenatal education; however, this discrepancy may reflect the BFH context of our study, where all mothers received standardized daily lactation consultations during hospitalization^18,19^.

Although prior studies suggested that higher LATCH scores might predict continued breastfeeding at 6 weeks, evidence regarding their predictive value for 6-month EBF has been limited^14,20^. Our results contribute to this evidence base; when analyzed as a continuous variable, the LATCH score was not independently associated with EBF at 6 months.

Certain practices emerged as stronger predictors of sustained breastfeeding. EBF within the first 48 h and the absence of bottle or pacifier use were significantly associated with maintaining EBF at 6 months. These findings are consistent with the study of Chantry et al., who showed that infants who avoided in-hospital formula and bottle exposure were more likely to continue breastfeeding. Our results reinforce the importance of avoiding pacifier or bottle introduction as a key strategy to protect breastfeeding continuity^21^.

In the multivariate analysis, bottle use was the only significant independent predictor of early cessation of EBF. This strong association highlights the disruptive role of bottle use on breastfeeding continuity and supports policies discouraging unnecessary bottle introduction in the early postpartum period. Although pacifier use did not remain significant in the multivariate model, its strong univariate association suggests it may still contribute to early EBF cessation and should also be discouraged as part of comprehensive breastfeeding support strategies.

The relatively high rate of 6-month EBF in our cohort (54.8%)—well above the national average in Türkiye—underscores the effectiveness of structured breastfeeding support services. Our hospital's Baby-Friendly policies, including daily lactation consultations and follow-up for mothers with low LATCH scores, likely contributed to these results. Recent United Nations Children's Fund (UNICEF) reports confirm that global EBF rates remain at only 48%, and the WHO/UNICEF 2030 goal of 70% is still distant^22,23^. These findings reinforce the need for hospital-based interventions, such as minimizing early bottle use and providing targeted lactation support, to bridge the gap between current practices and international targets^24^.

This study has limitations. It was conducted in a single BFH, which may limit generalizability, and the relatively high EBF rate may be influenced by structured lactation support typical of BFH settings; however, the absence of a non-BFH comparison group limits causal interpretation. Breastfeeding data relied on maternal self-reports, potentially introducing recall bias. Although 8.8% of dyads were lost to follow-up, the attrition rate remained low for a prospective study, but a small degree of selection bias cannot be entirely excluded, although such bias is unlikely to have meaningfully altered the findings. Despite these limitations, the multivariate models demonstrated acceptable fit and supported the interpretation of factors influencing 6-month EBF.

## CONCLUSION

Since bottle use is the most important factor affecting EBF at 6 months, preventing bottle use is a critical intervention in maintaining breastfeeding. Furthermore, the LATCH score is insufficient in predicting EBF in the long term. Standardized, validated follow-up mechanisms are also essential to ensure accurate long-term breastfeeding outcome assessment.

## Data Availability

The datasets generated and/or analyzed during the current study are available from the corresponding author upon reasonable request.
