# Supplementary material for: LATCH score and 6-month exclusive breastfeeding in a Baby-Friendly Hospital
Source: Rev Assoc Med Bras (1992). 2026 Jul 10;72(5):e20251466. doi: 10.1590/1806-9282.20251466 (PMC13361044; doi:10.1590/1806-9282.20251466)
Supplement: Supplementary Table 1 [file 1806-9282-ramb-72-05-e20251466-Suppl01.docx]

**Supplementary Table 1.** Binary logistic regression analysis of factors associated with exclusive breastfeeding for 6 months*.

|  | p-value | OR [Exp(b)] | 95%CI (lower) | 95%CI (upper) |
| --- | --- | --- | --- | --- |

| Maternal age 26–35 | 0.489 | 0.931 | 0.403 | 2.132 |
| --- | --- | --- | --- | --- |
| Maternal age ≥36 | 0.387 | 1.722 | 0.503 | 5.890 |
| Maternal education (high school) | 0.142 | 1.675 | 0.777 | 3.591 |
| Maternal education (university) | 0.076 | 2.275 | 0.917 | 5.639 |
| Initiation of breastfeeding (1 h) | 0.697 | 1.314 | 0.332 | 5.199 |
| Nutrition in the first 48 h | 0.227 | 1.792 | 0.697 | 4.608 |
| Pacifier use | 0.808 | 1.079 | 0.584 | 1.994 |
| Bottle use | **<0.001** | 16.672 | 8.875 | 31.318 |

*Model χ^2^ (df=8)=123.936, p<0.001, Nagelkerke R^2^=0.451, Overall classification accuracy=80.4%, Hosmer-Lemeshow p=0.941 (good fit). CI: confidence interval.
